# The prevalence and risk factors for depressive symptoms in frontline nurses under COVID-19 pandemic based on a large cross-sectional study using the propensity score-matched method

**DOI:** 10.1186/s12888-021-03143-z

**Published:** 2021-03-16

**Authors:** Hongyan Wang, Xiaoling Dai, Zichuan Yao, Xianqing Zhu, Yunzhong Jiang, Jia Li, Bin Han

**Affiliations:** 1 Department of Thoracic Surgery, Shengjing Hospital of China Medical University, Shenyang, China; 2 Department of Urology, Shengjing Hospital of China Medical University, No. 36, San Hao Street, Shenyang, 110004 Liaoning China

**Keywords:** Depressive symptoms, Nurse, Psychological capital, Sleep quality, depression

## Abstract

**Introduction:**

To explore the prevalence of depressive symptoms and the associated risk factors in frontline nurses under COVID-19 pandemic.

**Methods:**

This cross-sectional study was conducted from February 20, 2020 to March 20, 2020 and involved 562 frontline nurses. The effective response rate was 87.68%. After propensity score matched, there were 498 participants left. Extensive characteristics, including demographics, dietary habits, life-related factors, work-related factors, and psychological factors were collected based on a self-reported questionnaire. Specific scales measured the levels of sleep quality, physical activity, depressive symptoms, perceived organization support and psychological capital. Adjusted odds ratios and 95% confidence intervals were determined by binary paired logistic regression.

**Results:**

Of the nurses enrolled in the study, 50.90% had depressive symptoms. Three independent risk factors were identified: poor sleep quality (OR = 1.608, 95% CI: 1.384–1.896), lower optimism of psychological capital (OR = 0.879, 95% CI: 0.805–0.960) and no visiting friend constantly (OR = 0.513, 95% CI: 0.286–0.920).

**Conclusions:**

This study revealed a considerable high prevalence of depressive symptoms in frontline nurses during the COVID-19 outbreak, and identified three risk factors, which were poor sleep quality, lower optimism of psychological capital, and no visiting friend constantly. Protecting mental health of nurses is important for COVID-19 pandemic control and their wellbeing. These findings enrich the existing theoretical model of depression and demonstrated a critical need for additional strategies that could address the mental health in frontline nurses for policymakers.

## Introduction

Coronavirus disease 2019 (COVID-19), with an epicentre in Wuhan of China, has spread globally [1]. The World Health Organization Emergency Committee declared COVID-19 is an international public health emergency and outbreak in late January 2020 [2]. By May 1, 2020, there were 3,067,015 confirmed COVID-19 cases, including 214,375 associated deaths [2]. Such large-scale international public health threat presented severe challenges for medical staff. For example, frontline medical staff are under both physical and psychological pressure, staff members who experienced symptoms of depression were at increased risk of making errors in patient care [3]. Maintaining good mental health among medical staff is essential to prevent infectious disease spread and ensuring long-term wellbeing of staff [4]. Therefore, mental health of frontline medical staff should be supported during the outbreak of COVID-19.

Depression is characterized by low or absent positive feelings, and it can result in substantial impairments in social relationships, such as divorce, distancing from relatives or friends, limited social support, less success in the workplace, and illness [5]. Depression may also lead to suicidal ideation. Those who recover from depression still show impaired function in social and occupational domains [6]. As reported in the Global Burden of Disease study [7], the number of cases of depression worldwide increased from 172 million in 1990 to 258 million in 2017, representing a 49.86% increase during the study period. A survey of > 50,000 medical staff from Australia demonstrated an increased incidence of depression along with a two-fold increased incidence of suicidal ideation compared with the general population [8].

Nurses appear to be at an increased risk of developing mental disorders, which may reflect longer hours at work, separation from their families, and caring for a large number of patients [9]. Depression impairs both psychological and physical functions, which diminish professional performance, affects the quality of patient treatment outcomes, and provokes conflicts with patients, or colleagues [10]. Therefore, depression is a critical issue, not only for nurses themselves, but also for the health and safety of the patients they treat [11].

As a new an international public health emergency, COVID-19 has generated great research interest. However, data on the prevalence and risk factors of depressive symptoms among frontline nurses during the COVID-19 outbreak are none in China. Therefore, we developed a survey to determine the prevalence of depressive symptoms and its associated risk factors in this population.

## Methods

### Study design

This cross-sectional study was based on WeChat-based survey programme Questionnaire. From February 20, 2020 to March 20, 2020, a total of 641 frontline nurses responding to the COVID-19 outbreak participated in the present study. Data from participants who did not provide information on any of the variables of interest were excluded (*n* = 79). Overall, data from 562 participants were included in the final analyses. The average questionnaire spent 10 to 15 min.

### Inclusion and exclusion criteria

**Fig. 1:**
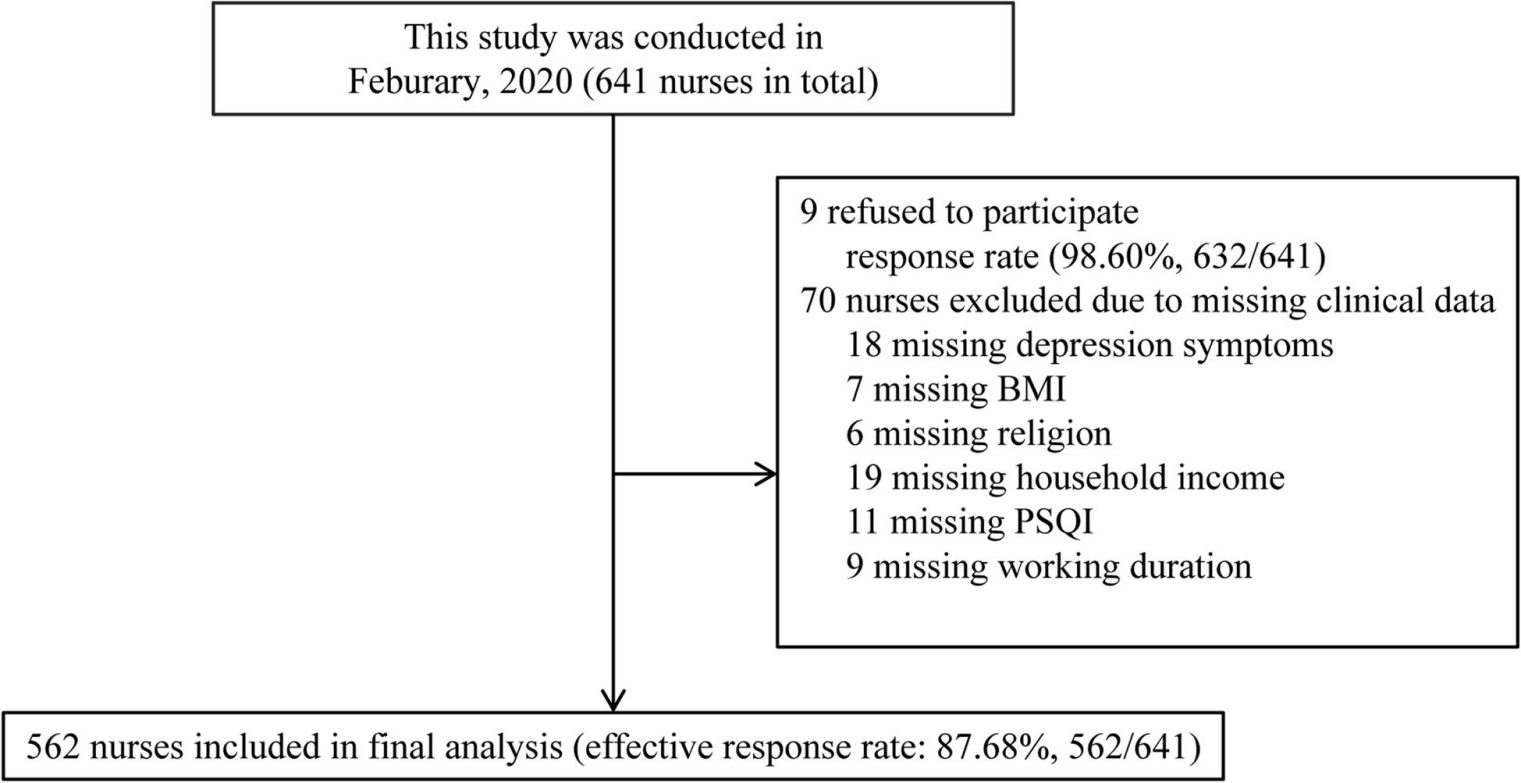
Flowchart of this study. Abbreviations: BMI, body mass index; PSQI, Pittsburgh sleep quality index

The inclusion criteria were as follows: occupationally active nurses who were employed in our hospitals. The following exclusion criteria were used: nurses who had participated in work less than 3 months or refused to participate in this program. Nurses who did not provide complete psychological questionnaire or clinical data were excluded either. Finally, effective responses were obtained from 562 individuals (effective response rate: 87.68%). A flow chart illustrating the process is detailed in Fig. 1.

### Measurement of characteristics

In this study, demographic characteristics included age, gender, BMI (kg/m^2^). Dietary habits included smoking status (current vs. never plus former), alcohol habit (current vs. never plus former), coffee habit (current vs. never plus former); Life related factors included sleep quality (PSQI, Pittsburgh sleep quality index scores), physical activity (IPAQ, International Physical Activity Questionnaire, Mets×hour/week), have religions (yes vs. no), marital status (single/ divorce/ separation/ widow vs. married /cohabitation), have siblings (yes vs. no), household income monthly (RMB, yuan) was categorized as,< 5000, ≧5000, < 10,000 and ≧10,000, experienced major life events (yes vs. no), visiting friend constantly (yes vs. no). Smoking habit was categorized as current smoker (≧ 1 cigarette per day and last ≧ 6 months), former smoker (stop smoking ≧ 6 months), and never smoker. Alcohol habit and coffee habit as categorized as current drinker (≧ 1 time per day and last ≧ 6 months), former drinker (stop drinking ≧ 6 months), and never drinker.

Work related factors included years of service (≤ 5 years vs. 5 years < and ≤ 10 years vs. > 10 years), specialty (surgery vs. internal medicine vs. obstetrics and gynecology vs. pediatrics vs. others), working duration (< 40 h/week vs. 40–60 h/week vs. > 60 h/week), and night shifts (times/month).

Psychological characteristics included Perceived Organization Support (POS) scale, Psychological Capital Questionnaire (PsyCap), and Patient Health Questionnaire (PHQ9).

Physical activity (PA) in the most recent week was assessed using the short form of the International Physical Activity Questionnaire (IPAQ) [12]. The questionnaire asked whether subjects had performed any activities from the following categories during the previous week: walking; moderate activity (household activity or child care); vigorous activity (running, swimming, or other sports activities). Metabolic equivalent (MET) hours per week were calculated using corresponding MET coefficients (3.3, 4.0, and 8.0, respectively) according to the following formula: MET coefficient of activity × duration (h) × frequency (days). Total PA levels were assessed by combining separate scores for different activities.

Sleep quality was measured by Pittsburgh sleep quality index (PSQI),which was developed by Buysse et al. [13]. It is a self-report on subjective sleep quality over the last 4 weeks with 18 questions. The first four questions enquire about times (bed time, number of minutes it took for the participant to fall asleep, get up time, and hours of sleep per night). The next 10 questions ask how often the participant had trouble sleeping because of different reasons (e.g. woke up in the middle of the night, need to go to the bathroom, cough, and bad dreams). Each of these questions must be answered on a 4-point scale ranging from “never” to “three times or more a week.” Additional questions include a subjective rating of the participants’ sleep quality (4-point scale from “very good” to “very bad”), the use of sleep medication, and trouble staying awake during the day (4-point scale ranging from “never” to “three times or more a week”). The final question asks if it has been a problem for the participant to keep up enough enthusiasm for getting things done (4-point scale ranging from “no problem at all” to “a very big problem”). The 18 items of the PSQI form seven-component score ranging from 0 to 3 (sleep quality, sleep latency, sleep duration, sleep efficiency, sleep disturbances, sleep medication, and daytime dysfunction) that can be summed up to a general score. Higher scores represent worse sleep quality. Poor sleep quality is indicated by total score of 6 or greater.

Experienced major life events included separation/divorce, death or serious illness of closely family members, serious injury/traffic accident, violence, unemployment, natural disasters, death or serious illness of partner, serious conflict with family, medical disputes, or income decrease/debt.

### Measurement of organization support

The Chinese version of the Perceived Organization Support Questionnaire (POS) was utilized to measure the level of organization support [14]. There were nine items, The score of each item is given on a 7-point Likert-type scale in accordance with the nurses’ personal experiences, ranging from 1 (very strongly disagree) to 7 (very strongly agree). The total score ranges from 9 to 63, with a higher score indicating higher social support. The POS has good reliability and validity among various Chinese.

### Measurement of psychological capital

PsyCap was evaluated by the Chinese version of the 24-item Psychological Capital Questionnaire (PCQ) [15]. The PCQ is comprised of four dimensions: self-efficacy (6 items), hope (6 items), resilience (6 items), and optimism (6 items). Each question is scored from 1 (strongly disagree) to 6 (strongly agree). Higher scores indicate higher levels of psychological capital. The PCQ has demonstrated adequate reliability and validity in multiple samples [16].

### Measurement of depressive symptoms

Depressive symptoms were measured by clinically validated scales for PHQ09. The PHQ09 scale comprises 9 items, and each item includes 4-point Likert-type scale responses that describe the frequency of subjects’ feelings in the past Two week ranging from 0 to 3. The summed score ranges from 0 to 27, with a higher score indicating more severe depressive symptoms. The presence of depressive symptoms was defined as a PHQ09 score ≧ 5. The Chinese PHQ09 scale has been widely used in previous studies [17].

### Propensity score-matching

**Fig. 2:**
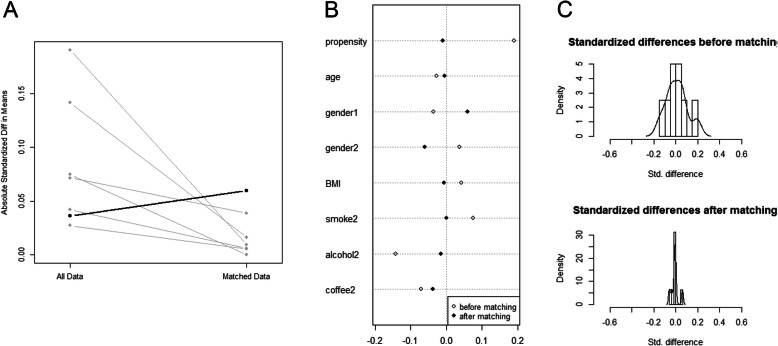
Plot of propensity score-matched in this study. **a** line plot foe individual differences of depressive symptoms. **b** dot-plot for standardized mean differences of depressive symptoms. **c** histogram for standardized mean differences (before and after) of depressive symptoms

We used the propensity score-matching (PSM) method to adjust baseline confounding variables between the depressive symptoms and non-depressive symptoms nurses in an effort to derive more accurate conclusions. Multivariate logistic regression analysis was used to determine propensity scores for each participant based on gender, age, body mass index, smoking status, alcohol habit, and drinking coffee habit, which were demographics or life habit. For assessing the calibration of the logistic regression model, the Hosmer–Lemeshow goodness-of-fit test (*P* = 0.83) was performed for this logistic regression model [a high *P* value (> 0.05) was interpreted as a good fit for the models]. Depressive symptoms and non-depressive symptoms groups were matched 1:1 by using a caliper width 0.2 of the standard deviation of the logit of the propensity score through the nearest neighbor matching, as this value minimized the mean squared error of the estimated treatment effect in several scenarios. (Fig. 2). The balance diagnostics completed for the propensity score method and showed no difference of covariates between two groups (|Standardized Difference| of all covariates was < 0.1) [18].

### Statistical analysis

Sample size calculation was based on the prevalence of depression (The proportion is 0.27) [19–21]. The 95% confidence interval width is 0.08, Type I error α was 0.05, two-tailed *P*-value was < 0.05, was calculated by PASS 11.0. We determined a minimum sample size of 496 participants.

Data were analyzed by SPSS 22.0 for Windows (SPSS Inc., Chicago, IL, USA). Continuous variables were presented as the median (interquartile range). Categorical variables were reported as the number (percentage). Univariate analysis was conducted by binary paired logistic regression model. Baseline variables that were considered clinically relevant or that had a *p*-value < 0.15 in the univariate analysis were included in a multivariate binary paired logistic regression model. Adjusted odds ratios and 95% confidence intervals (95% CI) were also determined after adjusting for potential confounders by binary paired logistic regression. Cutoff values and the area under the curve (AUC) for continuous variables which were independent risk factors for depressive symptoms were calculated through receiver operating characteristic curve analysis. A p-value of less than 0.05 was considered statistically significant.

## Results

There were 562 nurses included in this study finally (Fig. 1). The demographic characteristics, dietary habits, life related factors, work related factors, and psychological characteristics were displayed in Table 1. Of these nurses, they reported 50.90% (286) with depressive symptoms. The average age and BMI were 35.00 and 21.90 kg/m^2^, respectively. The most nurses were female gender, drinking alcohol, drinking coffee, married status, and never smoker, had no religion, no experienced major life events, ≧ 10,000 yuan/month household income, and 40–60 h/week working duration. The ratio visiting friends constantly, have siblings and years of service were balanced among each groups. The average of PSQI, PA, night shifts, POS (self-efficacy, hope, resilience, and optimism), and PsyCap, were also presented in Table 1 specifically.

**Table 1:** Characteristics of nurses before and after PSM by depressive symptoms in this study during the COVID-19 outbreak

Variables	Before PSM 562 (100%) nurses	After PSM 498 (100%) nurses
Depressive symptoms (yes)	286 (50.90)	1:1 PSM
**Demographic characteristics**		
Age (years)	35.00 (34.00, 36.00)	35.00 (34.00, 36.00)
Sex (male vs.female)	118 (21.00)/444 (79.00)	100 (20.10)/398 (79.90)
BMI (kg/m^2^)	21.90 (19.80, 24.23)	22.00 (19.97, 24.22)
**Dietary habits**		
Smoking habit (current yes)	8 (1.40)	4 (0.80)
Alcohol habit (current yes)	293 (52.10)	254 (51.00)
Coffee habit (current yes)	430 (76.50)	380 (76.30)
**Life related factors**		
Sleep quality (PSQI scores)	6.00 (4.00, 7.00)	6.00 (4.00, 7.00)
Physical activity (IPAQ Mets×hour/week)	9.90 (6.60, 23.73)	9.90 (6.60, 23.21)
Have religions (yes)	25 (4.40)	21 (4.20)
Marital status		
Single/divorce/separation/widow	232 (41.30)	213 (42.80)
Married/cohabitation	330 (58.70)	285 (57.20)
Have siblings (yes)	268 (47.70)	229 (46.00)
Household income (Yuan/month)		
< 5000	33 (5.90)	27 (5.40)
≧5000, < 10,000	121 (21.50)	111 (22.30)
≧10,000	408 (72.60)	360 (72.30)
Experienced major life events (yes)	180 (32.00)	162 (32.50)
Visiting friend constantly (yes)	259 (46.10)	231 (46.40)
**Work related factors**		
Years of service		
≤ 5 years	158 (28.10)	146 (29.30)
5 years < and ≤ 10 years	239 (42.50)	216 (43.40)
> 10 years	165 (29.40)	136 (27.30)
Speciality		
Surgery	68 (12.10)	60 (12.00)
Internal medicine	159 (28.20)	143 (28.70)
Obstetrics and Gynecology	61 (10.90)	54 (10.80)
Pediatrics	64 (11.40)	56 (11.20)
Others	210 (37.40)	185 (37.10)
Working duration (hours/week)		
40–60 h	409 (72.80)	357 (71.70)
< 40 h	18 (3.20)	14 (2.80)
> 60 h	135 (24.00)	127 (25.50)
Night shifts (times/month)	1.00 (1.00, 4.00)	1.00 (1.00, 4.00)
**Psychological characteristics**		
POS (scores)	42.00 (36.00, 52.00)	42.00 (36.00, 52.00)
PsyCap-efficacy (scores)	25.00 (22.00, 29.00)	25.00 (22.00, 29.00)
PsyCap-hope (scores)	25.00 (22.00, 29.00)	25.00 (22.00, 29.00)
PsyCap-resiliency (scores)	26.00 (23.00, 29.00)	26.00 (23.00, 29.00)
PsyCap-optimism (scores)	25.00 (22.00, 28.00)	25.00 (22.00, 28.00)

Continuous variables were reported median (interquartile range), categorical variables were reported as number (percentage)

*Abbreviations*: *COVID-19* coronavirus disease 2019, *PSM* propensity score matching, *BMI* body mass index, *PSQI*, Pittsburgh sleep quality index, *IPAQ* International Physical Activity Questionnaires, *POS* Perceived Organization Support, *PsyCap* Psychological Capital

A balance of baseline confounding variables between the two groups (depressive symptoms vs. non-depressive symptoms) after PSM was achieved (Fig. 2). There were 498 nurses left finally. In matched group, depressive nurses had higher score of PSQI, and lower scores of POS and PsyCap. They also had higher ratio of experienced major life events and 5 to 10 years of service. They had lower ratio of have siblings, married and visiting friends constantly. All above variables were statistical differences in univariate analysis and then included multivariate analysis, see details in Table 2.

**Table 2:** Univariate analysis of the risk factors for depressive symptoms of nurses before and after PSM during the COVID-19 outbreak

Variables	PSM before 562 nurses			PSM after 498 nurses		
	Depressive symptoms 286 nurses	Non-depressive symptoms 276 nurses	p	Depressive symptoms 249 nurses	Non-depressive symptoms 249 nurses	p
**Demographic characteristics**						
Age (years)	35.00 (34.00, 36.00)	35.00 (34.00, 36.00)	0.750	35.00 (34.00, 36.00)	35.00 (34.00, 36.00)	0.953
Sex (male vs.female)	58 (20.30)/228 (79.70)	60 (21.70)/216 (78.30)	0.671	53 (21.30)/196 (78.70)	47 (18.90)/202 (81.10)	0.378
BMI (kg/m^2^)	22.05 (19.80, 24.30)	21.85 (19.73, 23.98)	0.593	21.80 (19.80, 24.20)	21.60 (19.70, 23.90)	0.930
**Dietary habits**						
Smoking habit (current yes)	3 (1.00)	5 (1.80)	0.451	2 (0.80)	2 (0.80)	1.000
Alcohol habit (current yes)	159 (55.60)	134 (48.60)	0.095	128 (51.40)	126 (50.60)	0.715
Coffee habit (current yes)	223 (78.00)	207 (75.00)	0.406	192 (77.10)	188 (75.50)	0.663
**Life related factors**						
Sleep quality (PSQI scores)	7.00 (5.00, 9.00)	4.00 (3.00, 6.00)	< 0.001	7.00 (5.00, 9.00)	4.00 (3.00, 6.00)	< 0.001
Physical activity (IPAQ Mets×hour/week)	8.96 (6.60, 22.73)	11.55 (6.60, 26.55)	0.443	8.25 (6.60, 21.28)	11.55 (6.60, 26.40)	0.195
Have religions (yes)	16 (5.60)	9 (3.30)	0.185	12 (4.80)	9 (3.60)	0.493
Marital status			0.061			0.026
Single/divorce/separation/widow	157 (54.90)	173 (62.70)		119 (47.80)	94 (37.80)	
Married/cohabitation	129 (45.10)	103 (37.30)		130 (52.20)	155 (62.20)	
Have siblings (yes)	122 (42.70)	146 (52.90)	0.015	99 (39.80)	130 (52.20)	0.009
Household income (Yuan/month)			0.398			0.230
< 5000	18 (6.30)	15 (5.40)		16 (6.40)	11 (4.40)	
≧5000, < 10,000	65 (22.70)	56 (20.30)		58 (23.30)	53 (21.30)	
≧10,000	203 (71.00)	205 (74.30)		175 (70.30)	185 (74.30)	
Experienced major life events(yes)	103 (36.00)	77 (27.90)	0.040	93 (37.30)	69 (27.70)	0.020
Visiting friend constantly (yes)	105 (36.70)	154 (55.80)	< 0.001	91 (36.50)	140 (56.20)	< 0.001
**Work related factors**						
Years of service			0.023			0.068
≤ 5 years	65 (22.70)	93 (33.70)		59 (23.70)	87 (34.90)	
5 years < and ≤ 10 years	132 (46.20)	107 (38.80)		120 (48.20)	96 (38.60)	
> 10 years	89 (31.10)	76 (27.50)		70 (28.10)	66 (26.50)	
Speciality			0.646			0.702
Surgery	37 (12.90)	31 (11.20)		27 (10.80)	33 (13.30)	
Internal medicine	81 (28.30)	78 (28.30)		71 (28.50)	72 (28.90)	
Obstetrics and Gynecology	30 (10.50)	31 (11.20)		30 (12.00)	24 (9.60)	
Pediatrics	33 (11.50)	31 (11.20)		29 (11.60)	27 (10.80)	
Others	105 (36.80)	105 (38.10)		92 (36.90)	93 (37.30)	
Working duration (hours/week)			0.405			0.485
40–60 h	204 (71.30)	205 (74.30)		175 (70.30)	182 (73.10)	
< 40 h	9 (3.10)	9 (3.30)		7 (2.80)	7 (2.80)	
> 60 h	73 (25.50)	62 (22.50)		67 (26.90)	60 (24.10)	
Night shifts (times/month)	1.00 (1.00. 4.00)	1.00 (1.00. 4.00)	0.503	1.00 (1.00. 4.00)	1.00 (1.00. 4.00)	0.515
**Psychological characteristics**						
POS (scores)	39.00 (33.00, 48.00)	46.00 (38.00, 54.00)	< 0.001	39.00 (32.00, 47.00)	46.00 (38.00, 54.00)	< 0.001
PsyCap-efficacy (scores)	24.00 (21.00, 27.00)	28.00 (24.00, 30.00)	< 0.001	24.00 (21.00, 27.00)	27.00 (24.00, 30.00)	< 0.001
PsyCap-hope (scores)	24.00 (20.00, 27.00)	28.00 (24.00, 30.00)	< 0.001	24.00 (20.00, 27.00)	28.00 (24.00, 30.00)	< 0.001
PsyCap-resiliency (scores)	24.00 (21.00, 27.00)	27.00 (24.00, 30.00)	< 0.001	24.00 (22.00, 27.00)	27.00 (24.00, 30.00)	< 0.001
PsyCap-optimism (scores)	24.00 (21.00, 27.00)	27.00 (24.00, 30.00)	< 0.001	24.00 (21.00, 27.00)	27.00 (24.00, 30.00)	< 0.001

Continuous variables were expressed as median (interquartile range); categorical variables were reported asnumber (percentage). P value was analyzed by univariate paired conditional logistic regression

*Abbreviations*: *COVID-19* coronavirus disease 2019, *PSM* propensity score matching, *BMI* body mass index, *PSQI*, Pittsburgh sleep quality index, *IPAQ* International Physical Activity Questionnaires, *POS* Perceived Organization Support, *PsyCap* Psychological Capital

Based on multivariate paired logistic regression, three independent risk factors for depressive symptoms were identified, as follows: poor sleep quality (OR = 1.608), lower optimism of PsyCap (OR = 0.879), and no visiting friends constantly (OR = 0.513), see details in Table 3.

**Table 3:** Multivariate analysis of the risk factors for depressive symptoms of nurses before and after PSM during the COVID-19 outbreak

Variables	PSM before		PSM after	
	OR (95% CI)	p	OR (95% CI)	p
Sleep quality (PSQI scores)	1.491 (1.354, 1.640)	< 0.001	1.608 (1.384, 1.896)	< 0.001
Have siblings (no vs. yes)	1.743 (1.164, 2.610)	0.007	1.314 (0.558, 3.093)	0.531
Visiting friend constantly (yes vs. no)	0.601 (0.401, 0.903)	0.014	0.513 (0.286, 0.920)	0.025
PsyCap-hope (scores)	0.899 (0.850, 0.952)	< 0.001	0.988 (0.887, 1.099)	0.819
PsyCap-optimism (scores)	0.908 (0.853, 0.966)	0.002	0.879 (0.805, 0.960)	0.004

The odds ratio and 95% confidence interval were analyzed by multivariate paired logistic regression

*Abbreviations*: *COVID-19* coronavirus disease 2019, *PSM* propensity score matching, *OR* odds ratio, *CI* confidence interval, *PSQI* Pittsburgh sleep quality index, *PsyCap* Psychological Capital

**Fig. 3:**
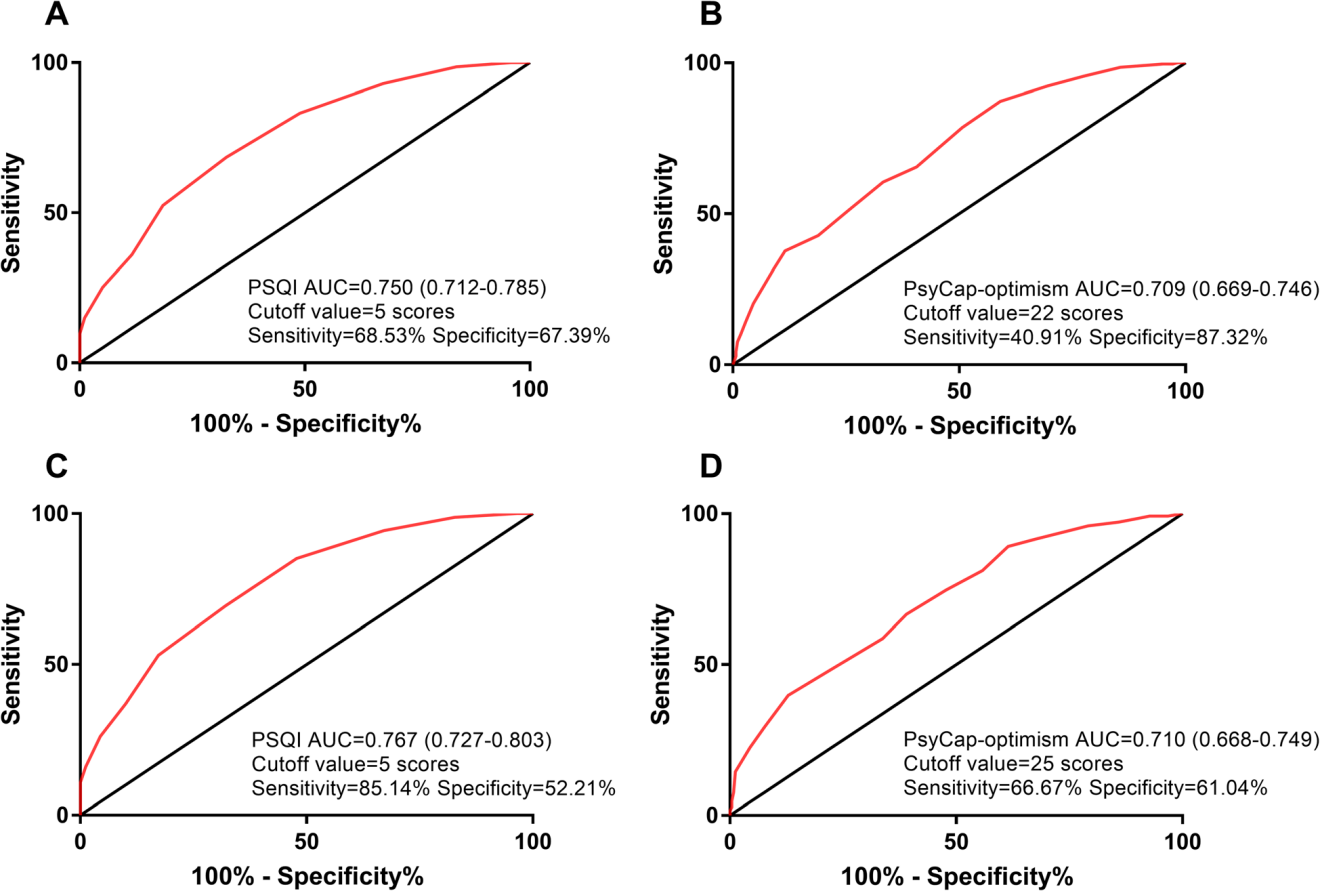
ROC curve and cutoff value for continuous independent risk factors of depressive symptoms before and after PSM. **a** PSQI scores of depressive symptoms before PSM. **b** Optimism of PsyCap scores of depressive symptoms before PSM. **c** PSQI scores of depressive symptoms after PSM. **d** Optimism of PsyCap scores of depressive symptoms after PSM. Abbreviations: ROC, receiver operating characteristic curve; AUC, area under the curve; PSQI, Pittsburgh sleep quality index; PsyCap, Psychological Capital; PSM, propensity score matching

In addition, the optimal cutoff point of continuous risk factors were analyzed by the receiver operating characteristic curve. The cutoff value for PSQI and optimism of PsyCap were 5 scores and 25 scores, see Fig. 3.

## Discussion

To the best of our knowledge, the present study is the first study to explore the prevalence and risk factors of depressive symptoms among frontline nurses responding to the COVID-19 outbreak in China. Although medical staff was not the main route of transmission in the case of COVID-19, they were in high-risk work environment. In addition, due to a large number of intensive care and emergency patients affected by this new infectious disease, therefore, emotional supports and encouragement for overcoming COVID-19 were urgently required. This study revealed a considerable high prevalence of depressive symptoms in this population, and there were three risk factors for depressive symptoms, which were poor sleep quality, lower optimism of psychological capital, and visiting friend rarely. In this study, 50.90% of nurses reported depressive symptoms. These results show that frontline nurses responding to the COVID-19 outbreak are almost more than ten times as likely to experience depressive symptoms compared to the general population, as demonstrated in a nationally representative face-to-face household survey in Germany between 2003 and 2008 (*n* = 5018, depressive symptoms rate = 5.6%) [22].

Loneliness, contrary to human nature due to the disposition toward social communication and unity is a negative situation occurring due to the insufficient quality and quantity of social relationship networks of an individual. Therefore, visiting friend constantly is usually a positive source of psychological supports. In line with this, visiting friend constantly was an independent factor for depressive symptoms in frontline nurses. Erzen et al. also found that loneliness as a negative emotion was considered to be a predisposing factor in depression in a meta analysis, which included 40,068 individuals [23].

This study demonstrated that poor sleep quality is positively associated with the prevalence of depressive symptoms in nurses. Previous studies have also shown that poor sleep quality is associated with a higher prevalence of depressive symptoms in different population, such as undergraduate students [24] and adolescents [25]. Harvey et al. [26] suggested that simply improving sleep can substantially reduce depressive symptoms. There are several mechanisms underlying the observed positive associations between poor sleep quality and the prevalence of depressive symptoms. First, sleep disturbance is associated with an increase in markers of systemic inflammation and pro-inflammatory cytokines produced by immune cells [27]. Immune signaling to the brain can lead to an exacerbation of the development of depressive symptoms in vulnerable individuals [28]. Second, serotonergic neurotransmission can interact with other brain areas modulating circadian rhythm and sleep [29]. In addition, the pathophysiology of depression is strongly linked to impairment in serotonin neurotransmission [30]. Third, previous research has suggested that circadian preferences may have an important role in the connection between sleep and depression [31].

Psychological capital (PsyCap) is a positive organizational behavior approach, which has been demonstrated as a positive resource of psychological capacities [15]. PsyCap consists of the four-dimensional resources, which are self-efficacy, hope, optimism, and resilience. Optimism includes the dispositional optimistic look towards the future. It has been reported that individuals with higher levels of PsyCap are able to have more confidence and make greater efforts to pursue success, and perceive positive expectations and attributes regarding consequences [32]. This study demonstrated that lower optimism was a significant risk factor for depressive symptoms in nurses. In line with this, Liu et al. [33] found that PsyCap could be a positive resource for combating depressive symptoms in physicians, which included 998 physicians in a cross-sectional survey of China. According to the study of Heinitz [34], optimism predicts depression. Hence, in a direct comparison, optimism appears to be the most relevant for depression of the four constructs.

There were several limitations in this study. First, this was a cross-sectional research that was unable to assess the causal relationships among study variables. Therefore, a longitudinal study should be carried out to verify our conclusions. Second, psycho-social variables were measured using self-report questionnaires, which might have recall and reporting bias. Third, depressive symptoms was confirmed by PHQ09, not a clinical diagnosis, which may influence the estimates. Since the questionnaire relies on patient self-report, all responses were not verified by the clinician. Moreover, diagnoses of Major Depressive Disorder or Other Depressive Disorder also require impairment of social, occupational, or other important areas of functioning and ruling out normal bereavement, a history of a Manic Episode (Bipolar Disorder), and a physical disorder, medication, or other drug as the biological cause of the depressive symptoms. Fourth, there were unmeasured confounding factors that contributed to the observed associations. Fifth, the participants of this study were recruited from one center. Nevertheless, this is the first study to explore the prevalence of depressive symptoms and its associated risk factors in frontline nurses during the COVID-19 outbreak from China.

## Conclusions

This study revealed a considerable high prevalence of depressive symptoms in frontline nurses during the COVID-19 outbreak, and identified three risk factors, which were poor sleep quality, lower optimism of psychological capital, and visiting friend rarely. Protecting mental health of nurses is important for COVID-19 pandemic control and their wellbeing. These findings enrich the existing theoretical model of depression and demonstrated a critical need for additional strategies that could address the mental health in frontline nurses for policymakers.

## Abbreviations

PSQI: Pittsburgh Sleep Quality Index
PHQ-9: Patient Health Questionnaire-9
OR: Odds ratio
CI: Confidence interval
BMI: Body mass index
IPAQ: International Physical Activity Questionnaire
MET: Metabolic equivalent
